# Incidence of Unapparent Preoperative Deep Vein Thrombosis in Patients with Traumatic Intraarticular Tibial Plateau Fracture

**DOI:** 10.3390/jcm14103490

**Published:** 2025-05-16

**Authors:** Henriette Hermel, Simon Yacoub, Firas Souleiman, Friederike Kohlmann, Andreas Kühnapfel, Christian Kleber, Katja S. Mühlberg, Ralf Henkelmann

**Affiliations:** 1 Department of Orthopedics, Trauma and Plastic Surgery, Leipzig University, Liebigstraße 20, 04103 Leipzig, Germany; simon.yacoub@medizin.uni-leipzig.de (S.Y.);; 2 Institute for Medical Informatics, Statistics and Epidemiology, Leipzig University, Haertelstrasse 16-18, 04107 Leipzig, Germany; 3 Department of Angiology, Leipzig University, Liebigstraße 20, 04103 Leipzig, Germany

**Keywords:** tibial plateau fracture, deep vein thrombosis, preoperative screening, vena cava filter, trauma, soft tissue injury, compartment syndrome, prophylactic anticoagulation

## Abstract

**Objective:** In patients with leg injuries, typical symptoms of thrombosis such as painful swelling may be misinterpreted as a consequence of the trauma. This has the potential to result in an unexpected embolism, especially during the perioperative period. This study investigates the incidence of unapparent preoperative deep-vein thrombosis (DVT) in patients with traumatic intraarticular tibial plateau fracture (TPF). A comprehensive analysis was conducted to identify possible risk factors, with particular attention paid to fracture severity and soft tissue injury. **Methods:** This retrospective single-centre study evaluated patient data from November 2021 to November 2024. It included 72 patients with traumatic intraarticular TPF who underwent surgery and received a preoperative compression ultrasonography screening. **Results:** The incidence of preoperative DVT was 23.6% (n = 17). Among these, 5.6% (n = 4) exhibited proximal thrombosis, while 18.1% (n = 13) demonstrated distal thrombosis. The fibular veins were predominantly affected (12/17). Patients with DVT suffered high-energy traumata, dislocations, compartment syndromes, and complex fractures (AO/OTA type C3: 82.4% vs. 52.7%) more often than patients without DVT and were more often immobilised with an external fixator. In 47.1% of DVT cases (n = 8), surgery could no longer be postponed, and an inferior vena cava filter was temporarily employed. The removal of the filter was successful in all cases, with no major complications encountered. **Conclusions:** This study reveals a high incidence (23.6%) of unapparent preoperative DVT in patients with traumatic intraarticular TPF despite prophylactic anticoagulation, particularly in those with severe fractures and soft tissue injuries. Systematic DVT screening and early anticoagulation are crucial to avoid potentially life-threatening complications.

## 1. Introduction

The tibial plateau is an integral part of the stability of the knee [1,2]. Although tibial plateau fractures (TPFs) are rare fractures (1%), the patient’s impairment can be devastating [3,4,5]. AO/OTA type C fractures are very unstable and often require surgical treatment (90%) to restore the anatomy and functionality of the joint [2,3,4].

Preoperative immobilisation of the affected leg is an essential part of soft tissue recovery. Inadequate soft tissue consolidation increases the risk of postoperative infection [5,6,7]. This can cause an elevated risk of developing deep vein thrombosis (DVT); thus, prophylactic anticoagulation is recommended [8,9,10]. Nevertheless, the incidence of preoperative DVT in patients with TPF ranges from 16 to 36% [11,12,13]. This range is broad and comparatively high in the context of DVT following trauma, where the incidence varies from 0.4–11% [14,15,16,17].

Immobilisation, trauma and hospitalisation are common risk factors for DVT [15]. DVT is known to increase morbidity and mortality, including subsequent pulmonary embolism (PE), especially in trauma patients [12,18,19].

Following a lethal index case of an unrecognised preoperative DVT after a TPF, a DVT screening algorithm was developed at our hospital. The aim of this study is to portray the incidence of unapparent preoperative DVT in patients with traumatic intraarticular TPFs and to reveal additional risk factors.

Our hypothesis is that intraarticular TPFs are associated with a unique high risk of DVT.

## 2. Materials and Methods

This retrospective single-centre study analysed data from November 2021 until November 2024. This study was approved by the Ethics Committee of the University of Leipzig (075/25-ek). Informed consent was waived according to paragraph 34 of the Saxon Hospital Law.

Eligible patients were identified using the hospital database with the International Classification of Diseases code for proximal tibia fractures (S82.11, S82.18). All data were manually reviewed to ensure correct coding and the application of our criteria.

Inclusion criteria were a recent TPF (<3 weeks old), a type B or C fracture (AO/OTA classification), age ≥ 18 years and an accomplished preoperative duplex compression ultrasound (DUS) [20].

Exclusion criteria were pathological fractures, definitive surgery in another hospital and conservative treatment.

After diagnosis of a TPF, soft tissue recovery was achieved via immobilisation (external fixator or splint).

Following the guidelines for thromboembolism prophylaxis, all patients received low molecular weight heparin (4500 IU Tinzaparin) [21]. Additionally, intermittent pneumatic compression was used.

**Figure 1:**
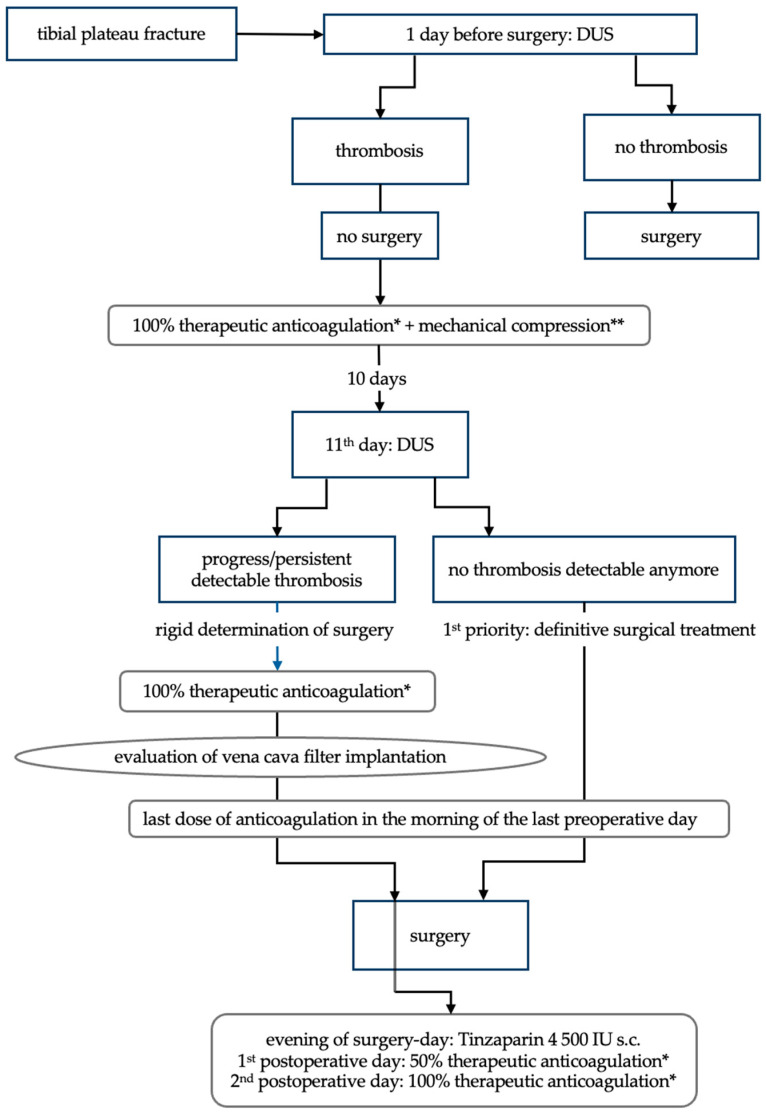
Screening and management of deep vein thrombosis in patients with tibial plateau fractures. DUS: duplex compression ultrasound; * 100% therapeutic anticoagulation: Tinzaparin 175 IU/kg body weight s.c.; IU/kg: international units per kilogram; s.c.: subcutaneously; Tinzaparin: low molecular weight heparin; ** mechanical compression in the form of a compression bandage, as far as there are no contraindications.

All patients with TPFs underwent DUS at least one day prior to surgery (see Figure 1). As a standard procedure, all deep and superficial veins were assessed for compressibility [9,22].

Once thrombosis was diagnosed, therapeutic anticoagulation was applied immediately, as well as compression bandaging, if possible. Because surgical treatment of a TPF should be performed as soon as the soft tissue conditions are adequate to achieve optimal results, thrombus resolution was reassessed after 10 days. If the thrombus had dissolved, the last preoperative dose of Tinzaparin was administered 24 h prior to surgery and continued after surgery according to the protocol (see Figure 1). If the thrombus had not dissolved, the perioperative implantation of a retrievable inferior vena cava (IVC) filter was evaluated in cooperation with the vascular specialists to allow timely surgery. Criteria for using an IVC filter were thrombus progression, severe thrombophilia or bleeding disorders. The filter was removed instantaneously after surgery. Postoperatively, the patients were mobilised according to standard procedures.

Regardless of thrombus resolution, patients with preoperative DVT received therapeutic anticoagulation and mechanical compression (usually knee stockings) for at least 3 months.

Patient characteristics and demographics were collected as shown in Table 1.

Common risk factors for venous thromboembolic events were enquired about, such as a history of venous thromboembolism (VTE), any known cancer or coagulopathies at the time of the incident and medical history including use of anticoagulants. To depict other diseases, the Charlson Comorbidity Index was calculated [23]. The American Society of Anesthesiologists (ASA) classification score was extracted from the premedication protocol [24].

Trauma-related data contained the dates of accident and admission, trauma mechanisms and affiliated classifications. High-energy trauma included traffic and bicycle accidents, falls from ≥1 m and accidents involving high external forces impinging on the body. Low-energy trauma was defined as falls during normal walking and falls from <1 m. Fracture-specific data included the AO/OTA classification and the soft-tissue injury classification by Tscherne/Oestern [20,25] (see Table 2).

Based on our working group’s criteria, concomitant injuries were categorised into three groups: none, not relevant (superficial soft tissue lesions, hematoma, abrasions) and relevant (e.g., compartment syndrome, further fractures) [26]. The type of preoperative immobilisation, the date and duration of definitive surgery and the length of stay (LOS) in the hospital were recorded.

DVT-related data comprised the location and extent of the detected thrombus. A thrombosis was defined as “distal” if the thrombus was below the popliteal vein and as “proximal” if the popliteal vein and veins above the popliteal level were affected. If the thrombus was in both locations, the thrombosis was considered proximal. Complications such as major intraoperative bleeding, symptomatic PE and, in the case of IVC filter use, complications according to its implantation and extraction were recorded.

Data were analysed using SPSS (version 30). Descriptive statistics are presented as absolute and relative frequencies, mean with standard deviation or median with interquartile range. For nominal data, Fisher’s exact test and the Freeman–Halton extension were used due to the small sample size. Ordinal data with very few categories were tested by nominal level. The Mann–Whitney U test was applied to account for the small sample size of the continuous data. The significance level was set at α = 0.05. Given the exploratory nature of our analysis, nominal *p*-values were reported without correction for multiple testing.

## 3. Results

### 3.1. Patient Cohort

**Figure 2:**
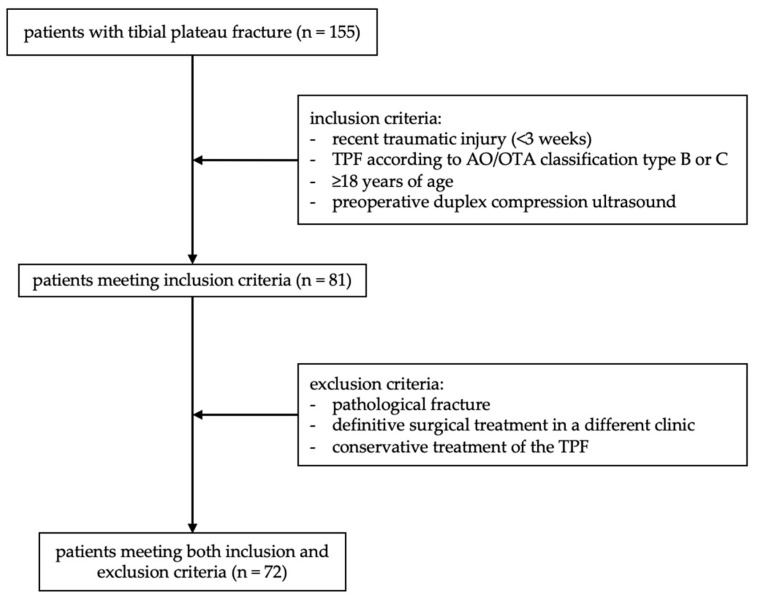
Process of patient selection.

A total of 155 patients with TPF were screened. Five patients with AO/OTA type A fractures, 14 minors and 55 patients without preoperative DUS were excluded. Of the remaining 81 patients, four underwent definitive surgery outside our hospital, and five received conservative treatment (see Figure 2).

Meeting all defined criteria, the final study cohort comprised 72 patients (m/f 32/40; mean age 52.3 ± 14.7; BMI median 25.6; interquartile range [IQR] = 7). Demographics are listed in Table 1.

### 3.2. DVT Patients’ Characteristics

**Figure 3:**
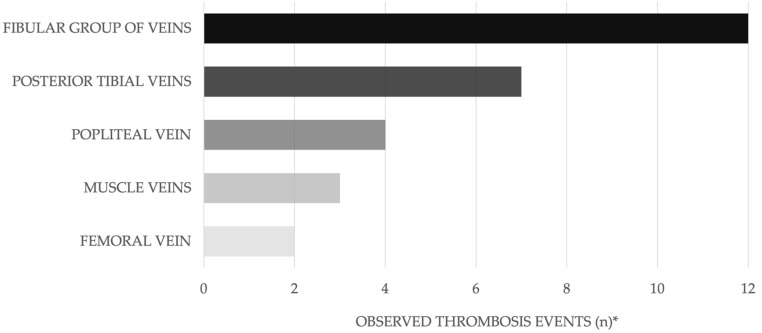
Location and extension of the thromboses. * Multiple venous segments may be affected concurrently in individual cases.

Seventeen patients (23.6%) had preoperative DVT. In the DVT group, 64.7% (11/17) were female. Four patients had proximal (5.6%) and thirteen had distal thrombosis (18.1%). The most frequently affected veins are shown in Figure 3.

Regarding patient characteristics, no significant differences between the groups regarding age, BMI, Charlson Comorbidity Index, abuse of noxae and relevant cardiovascular diseases were detected (see Table 1). We did observe comparatively more patients with ASA score III in the DVT group (17.6% vs. 9.1%), but this was not statistically significant (*p* = 0.58), as ASA score II was the most common in both groups.

Fracture-related data are presented in Table 2. DVT patients suffered from high-energy traumata (94.1% vs. 80%), open fractures (11.8% vs. 5.5%), bicondylar fractures (35.3% vs. 23.6%) and dislocations of the fracture (64.7% vs. 50.9%) more often. In the DVT group, 82.4% (14/17) were AO/OTA type C3 fractures compared with 50.9% (28/55) in the group without thrombosis (*p* = 0.12). The remaining fractures in the DVT group were all AO/OTA type B3 fractures (17.6%) compared with 36.4% B3 fractures in the group without thrombosis. Patients with preoperative DVT also tended to have higher grades of soft tissue injury and more frequent concomitant injuries, both on the same leg and in other body regions.

### 3.3. Known Risk Factors for DVT

Two patients with preoperative DVT presented with coagulopathies (one case of a diffuse bleeding tendency and factor XIII deficiency (antithrombotic) and one with a Factor V Leiden mutation (prothrombotic)).

None of the patients with preoperative DVT had a positive history of VTE, in contrast to the group without thrombosis (8/55). Most of them (6/8) were on long-term anticoagulation for this reason. No one had a positive cancer status.

Four patients with DVT (23.5%) presented with preoperative compartment syndrome, compared with only two patients (3.6%) in the group without thrombosis.

### 3.4. Perioperative Management of DVT Patients

Immobilisation was realised with an external fixator due to severe soft tissue damage in 82.4% (14/17) of the group with preoperative DVT, as compared with 63.6% (33/55) of the group without thrombosis (*p* = 0.15).

The interval from the day of injury to DUS was significantly longer in the group with preoperative DVT (median 8 vs. 5 days, *p* = 0.003). This resulted in prolonged immobilisation, and thus, in combination with postponed surgery, longer hospital stays for the DVT group, with a median of 29 days (IQR = 5) compared with 17 days (IQR = 10) for the group without thrombosis.

An IVC filter was implemented in eight patients with DVT (47.1%). The filter was removed without major complications in 100%. In one case, the removed IVC filter contained a thrombus (12.5%). No symptomatic PE or major intraoperative complications occurred. The presence of DVT did not significantly affect the duration of surgery (*p* = 0.69).

## 4. Discussion

At 23.6%, our study reveals a high incidence of unapparent preoperative DVT in patients with traumatic intraarticular TPFs. This observation ranks in the upper range of the preoperative DVT incidences reported in lower extremity fractures (4.9–30%) [12,27,28]. The fact that painful leg swelling can be a symptom of both fracture and thrombosis means that DVT diagnostics are not usually ordered, particularly as prophylactic anticoagulation is carried out as a standard procedure. However, the high incidence of unapparent DVT and the rate of 12.5% thrombus material retrieved in an IVC filter confirm the high thromboembolic risk of this cohort. In addition, trauma surgery already harbours a high thromboembolic risk on its own.

Trauma is known to induce a procoagulant state, and both trauma stress and systemic inflammatory response increase the risk of developing DVT [13,15,29]. According to Li et al., the more proximal the fracture of the lower extremity, the higher the risk of DVT [30]. Cai et al. found that the more proximal the tibial fracture, the higher the incidence of DVT [31]. These findings are consistent with our data. Four thromboses were located proximally (5.6%), the remaining 13 were isolated distal DVT. This aligns with the results by Wang et al. [12]. In 8/17 DVT patients, multiple veins were involved (see Figure 3).

Data from the Norwegian Venous Thrombosis Registry showed significant 5- and 10-year recurrence rates in patients with isolated distal DVT (14.7% and 27.2%, respectively) [32]. The cumulative incidence of recurrence was higher after unprovoked isolated distal DVT (72.7%) than after provoked (27.3%). 54.1% of registry participants had undergone surgery or had been immobilised 12 weeks preceding the thrombosis. During the 4.7 years of follow-up, patients experienced recurrent VTE in the form of PE (28.6%) and proximal DVT (33.3%).

Post-thrombotic syndrome occurs in 20–50% of DVT cases and is more common in proximal thromboses, which are also more likely to provoke PE [9,33]. This highlights the need for attentive screening, as 5.6% of our DVT patients had a proximal DVT. Fortunately, no symptomatic PE occurred in our cohort, and in one case a thrombus was captured by the implemented filter.

The process of thrombus maturation and resolution seems to be variable. Nevertheless, acute thrombi are at high risk for fragmentation and embolisation, whereas one-week old thrombi contain cross-linked fibrin and collagen and are more resistant and “organised” [34]. It remains unclear how the surgically unavoidable manipulation of a freshly thrombosed vein affects embolisation. However, it is undisputed that an early initiation of anticoagulation prevents thrombus propagation and reduces recurrence rates of VTE [35].

In contrast to the risk factors identified in previous studies, we observed a higher incidence of DVT in women [13,36,37,38]. Furthermore, although age is an independent risk factor for VTE, we did not observe a statistically significant difference between the groups, which corresponds to other studies’ findings [8,9,11,38]. In our dataset, age not differing significantly may be attributed to the, on average, young patient cohort.

**Figure 4:**
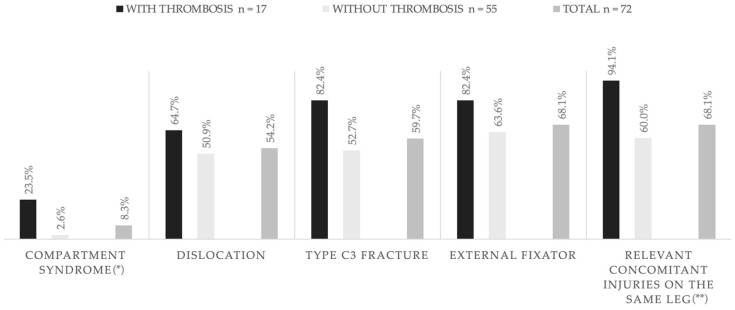
Comparison of the groups with and without thrombosis and the overall incidence, depicting frequencies of common features in patients with preoperative thrombosis. * *p* < 0.05, ** *p* < 0.01.

The assumption that the severity of the soft tissue injury affects the likelihood of developing a DVT can be partly confirmed. We do see a tendency of higher grades of soft tissue injury in closed fractures in the DVT group, yet this difference is not statistically significant (*p* = 0.19 for closed fractures). However, there were more compartment syndromes, more AO/OTA type C3 fractures (82.4% vs. 52.7%) and more dislocated fractures (see Table 2, Figure 4). This suggests an anatomical explanation for the development of DVT, as the morphology and dislocation of the fracture may impede venous blood flow. This is consistent with the fibular and posterior tibial veins being the most commonly affected veins, draining into the popliteal vein.

Further mutualities among the DVT group include more bicondylar fractures, open fractures and concomitant injuries both on the same leg and in other body regions. Only one patient with preoperative DVT had prothrombotic coagulopathy. Contrarily, some patients without preoperative DVT presented risk factors associated with its development. This corroborates the hypothesis of the fracture provoking the DVT.

The mean time from injury to DUS (or to diagnosis of DVT, respectively) differed by 3 days: 8 days in the thrombosis group (IQR = 7) versus 5 days in the group without thrombosis (IQR = 2). Hence, the DVT patients had been immobilised for 3 more days already when the DUS was performed. This could be partially attributed to a slightly longer stay in a different hospital prior to admission to ours (1.4 ± 2.1 days with DVT versus 0.9 ± 1.8 days without).

Due to the DVT, the time between injury and surgery was longer than for patients without DVT (20.1 vs. 8 days). This difference can be explained by our algorithm (see Figure 1), which aims to protect the patients from fatal complications following DVT. Furthermore, the preoperative conditioning of the soft tissue is essential for successful surgical management of TPF [6,7,13,39]. In addition to the gradual treatment of concomitant injuries, which was more prevalent in patients with DVT, this is a primary factor for surgical timing that cannot be shortened. However, delaying definitive surgical treatment is prone to impair the outcome, given that the optimal timing of surgery is determined 5–8 days after injury [39,40].

As anticipated, the overall LOS in the hospital was notably prolonged in the DVT group (29 vs. 17 days), with an additional 10 days solely due to DVT therapy. This entails a higher risk of infections, bleeding and muscle loss following inactivity, which impedes postoperative conditions. This in turn affects the outcome, as early mobilisation is crucial for restoring the knee joint and preventing thrombosis [5,39]. In addition, an extended hospital stay implicates economic consequences.

There is a known risk for perioperative bleeding under recent anticoagulation. Due to the heterogeneity of simultaneous issues that needed to be addressed, an increased demand for blood transfusions caused by recent anticoagulation remains unclear. Nonetheless, there were no significant bleeding events during surgery. Interestingly, the duration of surgery did not differ between the groups (*p* = 0.69, see Table 2).

In certain reports, the benefit of screening injured patients for DVT is called into question due to a lack of clinical significance of thrombosis, as many cases of DVT stay clinically occult [15,37,41]. However, treatment options for isolated distal DVT have not been conclusively studied, especially regarding the “wait and see” strategy. Furthermore, we do not have valid data on whether surgical manipulation of the vessels might loosen a clot and cause it to enter the system.

The reason for establishing our screening algorithm was a lethal outcome of such an unapparent DVT. The one thrombus captured in the IVC filter substantiates our approach. Therefore, the authors advocate for DUS as a noninvasive screening procedure involving little effort as compared with missing a thrombus that might kill the patient.

As a result of our study, we see our hypothesis confirmed that patients with traumatic intraarticular TPF and severe soft-tissue damage have an increased risk of unapparent DVT, probably due to the mechanism of injury and the anatomical proximity to the vessels. Neither thromboembolic nor bleeding complications occurred under full therapeutic anticoagulation according to our screening algorithm.

### Limitations

The retrospective character of the study precludes collection of additional data, including variables that may influence the patient’s risk of developing DVT, such as family history of DVT or the use of hormonal contraceptives/replacement therapy.

In favour of the comparability among the patients, concomitant injuries and injury mechanisms were simplified despite differences with corresponding impact on e.g., LOS.

The patient cohort is rather small; hence, these data allow us to assume correlation but not causation. An adequately powered prospective study would enable us to reliably analyse risk factors.

## 5. Conclusions

These data reveal a uniquely high incidence (23.6%) of preoperative unapparent DVT in patients with traumatic intraarticular TPF despite prophylactic anticoagulation. Female sex; high-energy trauma; severe injury of the tibial plateau, especially compartment syndrome and the use of an external fixator seem to affect the likelihood of DVT. Systematic DVT screening and early anticoagulation are crucial to prevent life-threatening complications. Our findings recall the discussion about surgical timing and in particular, the question as to whether an earlier TPF surgery with a higher risk of infection would outweigh the increased risk of thrombosis with extended hospitalisation.

## Figures and Tables

**Table 1:** Demographic and descriptive data.

	Unit/Specification	Thrombosis n = 17	No Thrombosis n = 55	Total n = 72	*p*-Value
Age	years	53.1 ± 14.7	52.1 ± 14.9	52.3 ± 14.7	0.65
BMI	kg/m^2^	24.5 [6]	26 [8]	25.6 [7]	0.17
Charlson Comorbidity Index *		1.5 ± 1.2	1.6 ± 1.5	1.5 ± 1.4	
Sex	**female**/male	11/6 (**64.7**/35.3)	**29**/26 (**52.7**/47.3)	40/32 (**55.6**/44.4)	0.39
Weight class	underweight	1 (5.9)	0 (0)	1 (1.4)	0.44
	normal weight	10 (58.8)	24 (43.6)	34 (47.2)	
	overweight	4 (23.5)	14 (25.5)	18 (25)	
	obesity I	1 (5.9)	9 (16.4)	10 (13.9)	
	obesity II	1 (5.9)	5 (9.1)	6 (8.3)	
	obesity III	0 (0)	3 (5.5)	3 (4.2)	
ASA classification score	I	2 (11.8)	8 (14.5)	10 (13.9)	0.58
	II	12 (70.6)	42 (76.4)	54 (75)	
	III	3 (17.6)	5 (9.1)	8 (11.1)	
Patient characteristics					
	**thromboembolic events in the past**	**0 (0)**	**8 (14.5)**	8 (11.1)	0.19
	**cancer**	**0 (0)**	**0 (0)**	0 (0)	n.a.
	**coagulopathy**	**2 (11.8)**	**0 (0)**	2 (2.8)	0.053
	long-term anticoagulant medication intake	0 (0)	6 (10.9)	6 (8.3)	0.33
	Diabetes mellitus	1 (5.9)	7 (12.7)	8 (11.1)	0.67
	arterial hypertension	5 (29.4)	21 (38.2)	26 (36.1)	0.51
	dyslipidemia	2 (11.8)	6 (10.9)	8 (11.1)	1
	alcohol abuse	1 (5.9)	3 (5.5)	4 (5.6)	1
	drug abuse	0 (0)	3 (5.5)	3 (4.2)	1
	nicotine abuse	3 (17.6)	15 (27.3)	18 (25)	0.53

Data are presented as n (%), mean ± standard deviation or median [interquartile range]. BMI = body mass index; ASA = American Society of Anesthesiologists; n.a. = not applicable because no events occurred in either group. * Analysed as a categorial variable but in favour of simplification, shown as mean with standard deviation.

**Table 2:** Characteristics of the injuries and their treatments.

	Unit/Specification	Thrombosis n = 17	No Thrombosis n = 55	Total n = 72	*p*-Value
**time from injury to surgery**	days	**20.1 ± 3.9**	**8 ± 3.5**	9 ± 9	**<0.001**
**time from admission to surgery**	days	**18.8 ± 3.9**	**7.1 ± 3.5**	9.85 ± 6.1	**<0.001**
**time from injury to DUS**	days	**8 [7]**	**5 [2]**	6 [3]	**0.003**
**time from DUS to surgery**	days	**13 [7]**	**1 [1]**	1 [5]	**<0.001**
duration of surgery	minutes	202 ± 48	199 ± 69	200 ± 64	0.69
**LOS in hospital**	days	**29 [5]**	**17 [10]**	19 [15]	**<0.001**
mechanism of trauma	**high-energy**	16 (**94.1**)	44 (**80**)	60 (83.3)	0.27
	low-energy	1 (5.9)	11 (20)	12 (16.7)	
transfer from a different hospital		10 (58.8)	30 (54.5)	40 (55.6)	0.76
**dislocation**		11 (**64.7**)	28 (**50.9**)	39 (54.2)	0.32
site of injury	right	8 (47.1)	24 (43.6)	32 (44.4)	0.80
	left	9 (52.9)	31 (56.4)	40 (55.6)	
AO/OTA type	B1	0 (0)	4 (7.3)	4 (5.6)	0.21
	B3	3 (17.6)	20 (36.4)	23 (31.9)	
	C1	0 (0)	2 (3.6)	2 (2.8)	
	**C3**	14 (**82.4**)	29 (**52.7**)	43 (59.7)	
open versus closed fracture	**open**	2 (**11.8**)	3 (**5.5**)	5 (6.9)	0.59
	closed	15 (88.2)	52 (94.5)	67 (93.1)	
**soft tissue injury** (**open** fracture)	II	1 (5.9)	2 (3.6)	3 (4.2)	1
	**III**	1 (**5.9**)	1 (**1.8**)	2 (2.8)	
soft tissue injury (**closed** fracture)	I	5 (29.4)	22 (40)	27 (37.5)	0.19
	II	8 (47.1)	27 (49.1)	35 (48.6)	
	**III**	2 (**11.8**)	1 (**1.8**)	3 (4.2)	
	no information	0 (0)	2 (3.6)	2 (2.8)	
affected condyles	unicondylar	11 (64.7)	42 (76.4)	53 (73.6)	0.36
	**bicondylar**	6 (**35.3**)	13 (**23.6**)	19 (26.4)	
**compartment syndrome**		4 (**23.5**)	2 (**3.6**)	6 (8.3)	**0.025**
**concomitant injuries on the same leg** *	none	1 (5.9)	22 (40)	23 (31.9)	**0.008**
	not relevant	0 (0)	0 (0)	0 (0)	
	relevant	16 (**94.1**)	33 (**60**)	49 (68.1)	
**concomitant injuries in other body regions** *	none	10 (58.8)	38 (69.1)	48 (66.7)	0.61
	not relevant	2 (**11.8**)	4 (**7.3**)	6 (8.3)	
	relevant	5 (**29.4**)	13 (**23.6**)	18 (25)	
form of immobilisation	**external fixator**	14 (**82.4**)	35 (**63.6**)	49 (68.1)	0.15
	splint	3 (17.6)	20 (36.4)	23 (31.9)	

Data are presented as n (%), mean ± standard deviation or median [interquartile range]. DUS = duplex compression ultrasound; LOS = length of stay. * Concomitant injuries were classified as none, not relevant (e.g., superficial soft tissue lesions, hematoma, abrasions) and relevant (e.g., further fractures).

## Data Availability

The collected data are available upon request.

## Abbreviations

The following abbreviations are used in this manuscript:

ASA	American Society of Anesthesiologists
BMI	Body mass index
DUS	Duplex compression ultrasound
DVT	Deep vein thrombosis
IU	International units
IVC	Inferior vena cava
LOS	Length of stay
MDPI	Multidisciplinary Digital Publishing Institute
PE	Pulmonary embolism
SPSS	Statistical Package for the Social Sciences
TPF	Tibial plateau fracture
VTE	Venous thromboembolism
